# Usabilidade de autoteste de antígeno para COVID-19: fatores associados e desafios relatados

**DOI:** 10.1590/0034-7167-2025-0197

**Published:** 2026-08-03

**Authors:** Isadora Silva de Carvalho, Lariane Angel Cepas, Talita Morais Fernandes, Carlos Arterio Sorgi, Ana Flávia Nacif Pinto Coelho Pires, Álvaro Francisco Lopes de Sousa, Isabella Silva de Carvalho, Ana Paula Morais Fernandes

**Affiliations:** I Universidade de São Paulo, Escola de Enfermagem de Ribeirão Preto. Ribeirão Preto, São Paulo, Brazil; II Instituto Superior de Lisboa e Vale do Tejo. Lisboa, Lisboa, Portugal; III Faculdade de Filosofia Ciências e Letras. Ribeirão Preto, São Paulo, Brazil; IV Universidade de Brasília. Brasília, Distrito Federal, Brazil; V Universidade Federal do Mato Grosso do Sul. Três Lagoas, Mato Grosso do Sul, Brazil; VI Escola Nacional de Saúde Pública. Lisboa, Lisboa, Portugal; VII Universidade Estadual Paulista “Júlio de Mesquita Filho”. Jaboticabal, São Paulo, Brazil

**Keywords:** Self-Testing, COVID-19, Rapid Diagnostic Tests, Health Services Accessibility, Vulnerable Populations., Autodiagnóstico, COVID-19, Pruebas Diagnósticas Rápidas, Accesibilidad a los Servicios de Salud, Poblaciones Vulnerables.

## Abstract

**Objectives:**

to assess the usability of a COVID-19 antigen self-test and identify factors associated with the perceived ease of use.

**Methods:**

cross-sectional study conducted in São Paulo (SP), Brazil, from June to July 2023, with 90 participants who used the self-test and completed a structured questionnaire. Descriptive, bivariate, and multivariable logistic regression analyses were performed.

**Results:**

only 33 participants (36.7%) reported high usability. Having an income above two minimum wages was significantly associated with a higher likelihood of rating the test as easy to use (ORa = 1.92; 95% CI: 1.15-5.67; p = 0.009). The main difficulties involved understanding the instructions (70%) and handling components such as the swab and buffer (45%).

**Conclusions:**

although promising for expanding access to diagnosis, the self-test faces barriers related to understanding and handling, especially among lower-income individuals. These findings reinforce the importance of clear instructions and technological adaptations tailored to users’ profiles.

## RESUMEN

**Objetivos:**

evaluar la usabilidad de una autoprueba de antígeno para COVID-19 e identificar los factores asociados con la percepción de facilidad de uso.

**Métodos:**

estudio transversal realizado en São Paulo (SP), Brasil, entre junio y julio de 2023, con 90 participantes que utilizaron la autoprueba y respondieron un cuestionario estructurado. Se realizaron análisis descriptivos, bivariados y regresión logística multivariada.

**Resultados:**

solo 33 participantes (36,7%) reportaron alta usabilidad. Tener ingresos superiores a dos salarios mínimos se asoció significativamente con una mayor probabilidad de evaluar la prueba como fácil de usar (ORa = 1,92; IC95%: 1,15-5,67; P = 0,009). Las principales dificultades se relacionaron con la comprensión de las instrucciones (70%) y el manejo de componentes como el hisopo y el tampón (45%).

**Conclusiones:**

aunque la autoprueba es prometedora para ampliar el acceso al diagnóstico, enfrenta barreras relacionadas con la comprensión y el manejo, especialmente entre personas con menores ingresos. Estos hallazgos refuerzan la importancia de contar con instrucciones claras y adaptaciones tecnológicas acordes con el perfil de los usuarios.

## INTRODUCTION

The Coronavirus Disease 2019 (COVID-19) pandemic, declared in 2020, had a profound and lasting impact on global public health. By the end of the main international emergencies in 2023, the world had recorded more than 700 million confirmed cases and approximately 7 million deaths attributed to the disease^(1)^. Brazil was among the most affected countries, with more than 37 million cases and over 700,000 deaths, reflecting not only the virulence of SARS-CoV-2 but also structural weaknesses in the health system and social inequality^(2)^.

In the Brazilian context, several strategies were implemented to contain the spread of COVID-19, such as awareness campaigns promoting frequent hand hygiene, social distancing, mask use in public spaces, and large-scale vaccination campaigns. However, this latter strategy became established as the main preventive measure only from 2021 onward. In the initial period of the pandemic, when vaccines were not yet available to the entire population, mass testing of symptomatic or exposed individuals played a key role in controlling transmission. Diagnostic testing enabled the rapid identification of cases, early isolation of infected individuals, and contact tracing, all of which were essential strategies to mitigate the spread of the disease, particularly in a scenario of high transmissibility and community circulation of the virus.

In this sense, among the measures adopted, expanded access to rapid antigen tests stood out by enabling quick diagnosis at lower cost and without the need for complex laboratory infrastructure^(3)^. Subsequently, the introduction of self-tests, authorized in Brazil by the National Health Surveillance Agency (ANVISA) in 2022, represented an important advance by allowing users to perform the test at home in a practical, confidential, and autonomous manner^(4)^. This measure is understood to have expanded access to diagnosis, especially among populations with mobility constraints or those that have historically faced barriers in accessing health services^(5,6)^.

However, despite the progress represented by the authorization of self-tests, their large-scale adoption in Brazil still faces important challenges^(7)^. Unlike tests performed in laboratory settings or under the supervision of health professionals, the use of self-tests assumes that individuals are able to understand the instructions provided, correctly perform all steps of the procedure, and adequately interpret the result^(8,9)^. Although this autonomy can enhance access to diagnosis, it also imposes additional barriers, especially for population groups with lower levels of education, limited health literacy, or restricted access to quality information.

Previous experiences with other types of self-tests, such as HIV diagnostic self-tests and fingerstick blood glucose tests for diabetes monitoring, show that difficulties related to understanding instructions, technically performing procedures, and interpreting results are not exclusive to the COVID-19 context^(8,10)^. Studies on HIV self-testing in Brazil, for example, report that participants often have questions about how to use the kit, fear making mistakes during the procedure, and feel insecure when faced with positive results, especially in the absence of professional guidance or accessible educational materials^(11,12)^. Similarly, users of glucometers report obstacles related to handling the device, correctly collecting the blood sample, and understanding measured values, particularly among older adults or individuals with low educational attainment^(13)^.

This evidence suggests that although self-tests expand autonomy and the reach of diagnostic strategies, their effectiveness depends directly on the support provided to users, the clarity of instructions, and the alignment of materials with the population’s sociocultural profile. Persistent difficulties may compromise both the accuracy of results and users’ confidence and adherence to home testing, underscoring the importance of public policies and instructional materials adapted to Brazilian sociocultural realities^(13)^.

This scenario has been especially challenging for several historically marginalized population groups, such as lesbian, gay, bisexual, transgender, queer, intersex, asexual, pansexual, and other identities (LGBTQIAP+), as well as sex workers, who already face structural barriers in accessing education, health, and decent work, as outlined in the Sustainable Development Goals (SDGs), particularly Goal 8, and who are marked by stigma, discrimination, and economic insecurity^(14)^. For these groups, the adoption of self-testing, although promising, may be even more complex due to limited access to clear information, technical support, and instructional material adapted to their realities^(15,16)^.

During the most critical years of the pandemic, these populations were more exposed to the risk of infection, often because they worked in informal jobs and were unable to adopt effective distancing measures^(17)^. In addition, the spread of misinformation and low health literacy hindered adherence to public health guidance and the correct use of diagnostic devices such as self-tests^(18-20)^.

Several studies have highlighted the importance of self-tests as complementary tools to formal diagnostic systems^(21,22)^. However, they have also pointed out limitations related to understanding instructions, the complexity of handling materials, and the reading of results, especially among individuals with low educational levels or limited access to information^(23,24)^.

Given the growing interest in expanding the use of self-tests for various health conditions, it is essential to understand the barriers faced by users in order to ensure not only the effectiveness of these devices but also their acceptability and adherence to their use. Nonetheless, a systematized review of the literature reveals a scarcity of national studies assessing self-test usability in real-world contexts, particularly among vulnerable populations. Analyzing these experiences can generate relevant evidence to improve implementation strategies and contribute to expanding the effective use of self-tests in the diagnosis of other health conditions beyond COVID-19.

## OBJECTIVES

To assess the usability of a COVID-19 antigen self-test and identify factors associated with the perceived ease of use.

## METHODS

### Ethical Aspects

The study was approved by the Research Ethics Committee of the Faculty of Philosophy, Sciences and Letters of Ribeirão Preto (CAAE: 51994921.7.0000.5407), and all participants provided written informed consent.

### Study Design

This was a cross-sectional study with an analytical approach, whose design and description followed the recommendations of the Strengthening the Reporting of Observational Studies in Epidemiology (STROBE) checklist.

### Study Setting

Data collection was carried out in the city of São Paulo (SP), between June and July 2023, a period marked by the consolidation of COVID-19 vaccination in Brazil and the expansion of rapid testing policies in the population. The study occurred within the “*USP na Comunidade*” (“USP in the Community”) initiative, promoted by the Office of the Vice Provost for Culture and University Extension of the University of São Paulo (USP), in partnership with the Joint United Nations Programme on HIV/AIDS in Brazil (UNAIDS). This initiative, aimed at promoting health and citizenship, uses Mobile Units to offer services, educational activities, and guidance to the population in public spaces with high foot traffic.

The Latin America Memorial was chosen as the site for the study due to its strategic location, high circulation of diverse audiences, and well-established tradition of hosting education, citizenship, and public health initiatives. Located in an easily accessible area of São Paulo, the Memorial receives a heterogeneous public on a daily basis, including students, workers, families, and groups in situations of social vulnerability. This diversity of profiles helped ensure a varied and representative sample, which was essential for evaluating the usability of self-tests across different population segments. In addition, the Memorial’s recognition as a welcoming and trusted space facilitated participant engagement and the integration of the study with the educational and health promotion campaigns conducted during the event.

### Population and Sample

Non-probability sampling was used, combining convenience sampling and a snowball strategy, in which initial participants were invited directly at the study site and could subsequently refer other potentially eligible individuals interested in participating and testing themselves on-site^(25)^. Individuals aged 18 years or older, residing in Brazil and with an apparent ability to understand and communicate adequately to respond to the face-to-face interview were included. Exclusion criteria were refusal to participate or inability to complete the questionnaire. The final sample consisted of 90 participants, a number defined by the recruitment dynamics during the study period. Despite the convenience-based nature of the sample, a post hoc power analysis was conducted to justify its adequacy.

For this analysis, a significance level (alpha) of 5%, statistical power (1-β) of 80%, a minimum expected odds ratio of 2.5 for the association between sociodemographic factors and high test usability, and a prevalence of high usability estimated at approximately 30% of participants were considered. Under these conditions, the sample of 90 individuals was sufficient to detect associations of moderate magnitude with reasonable statistical confidence, according to calculations performed using G*Power software, version 3.1.

### Study Protocol

Data collection was carried out through individual face-to-face interviews conducted by previously trained professionals. The main outcome of the study was the “usability of the COVID-19 antigen self-test”, defined as the extent to which a user, under real-world conditions, is able to understand the instructions, correctly perform the procedure, and interpret the result autonomously, safely, and satisfactorily. This concept was grounded in international literature and adapted to the context of the present study, considering the recommendations of Resolution of the Collegiate Board (RDC) No. 595/2022 of ANVISA^(26)^ and protocols validated by Bien-Gund^(27)^.

For data collection, a structured questionnaire was used, developed based on ANVISA guidelines (RDC No. 595/2022) and international usability assessment instruments. The questionnaire was composed of four sections:

1. Sociodemographic characteristics - including age, gender identity, sexual orientation, educational level, occupation, monthly family income, children, place of birth, and length of residence in the city/state.
2. Previous knowledge about COVID-19 and self-tests - addressing, for example, whether the participant had previously used any of these tests.
3. Self-test usability - including items related to understanding the instructions, handling components (swab, buffer, dropper), performing the procedure, and reading the result. Responses were predominantly dichotomous (yes/no), and this section also included an open-ended field for participants to describe, in their own words, any specific difficulties encountered during testing.
4. Overall satisfaction with the self-test - assessing the perceived usefulness of the instructions, suggestions for improvement, and willingness to recommend the test to others.

The usability section of the instrument was previously tested through a pilot test with a group of five participants from the target population to ensure clarity, relevance, and adequacy of the items in relation to the usability concept.

The study adopted a quantitative approach, using a structured questionnaire composed of 22 closed-ended questions, 21 of which had a binary format (yes/no), aimed at identifying practical and informational barriers in the self-testing process. In cases where participants indicated having faced any difficulty, an additional field was provided to describe the type of difficulty reported, the content of which was used only for descriptive purposes, without systematic qualitative analysis.

To quantify usability, a score was created, composed of 21 binary variables reflecting the absence of difficulties (“no” responses) at different stages of the self-test, with 1 point assigned to each response indicative of ease. Additionally, a practicality rating provided by the participant was included, ranging from 1 (not at all practical) to 5 (very practical), resulting in a total score from 0 to 26 points, with higher values indicating greater perceived usability. The cutoff point for classification into “high usability” versus “medium or low usability” was defined based on the sample median, a strategy frequently employed in exploratory studies when validated cutoff points are not available in the literature. The internal consistency of the score was assessed using Cronbach’s alpha (obtained value: 0.81), indicating good internal reliability of the construct.

### Statistical analysis

Statistical analyses were performed using R software, version 4.1.3. Initially, a univariate descriptive analysis was conducted, with calculation of absolute and relative frequencies for categorical variables and measures of central tendency and dispersion for continuous variables. Missing or omitted values were identified and treated as missing data, without imputation. For the bivariate analysis, association tests were used between the dependent variable, self-test usability (classified as a binary variable: 1 = high usability; 0 = medium or low), and independent sociodemographic variables. Associations were evaluated using Pearson’s chi-square test or, when necessary, Fisher’s exact test (p < 0.05).

The independent variables included social and demographic variables. All variables were previously dichotomized for the purposes of multivariable analysis. Next, a binary logistic regression model was fit with the outcome “high test usability”. Variables with p < 0.20 in the bivariate analysis were included in the multivariable model. The results were expressed as adjusted odds ratios (aOR) with 95% CI and p-values. Model fit quality was assessed using the likelihood ratio test (p < 0.01), as well as Cox-Snell (0.27), Nagelkerke (0.49), and McFadden (0.39) pseudo-R^2^ coefficients.

In addition, the ROC (Receiver Operating Characteristic) curve was used to evaluate the discriminative power of the final model, resulting in an area under the curve (AUC) of 0.86, indicating strong predictive ability.

## RESULTS

A total of 90 participants took part in the study. The mean usability score was 12.0 points (out of a theoretical maximum of 26), suggesting that, overall, users rated the self-test as moderately usable. The median score of 13 indicates that half of the participants reached this level of usability, which is consistent with a classification of medium usability. Considering the adopted cutoff point (median score), 33 participants (36.7%; 95% CI: 26.6%-47.8%) were classified as having high usability of the COVID-19 antigen self-test.

Approximately one-third of the participants reported high usability, while a smaller proportion experienced low usability. The presence of participants with the minimum score (0) shows that specific challenges in understanding and performing the self-test remain, reinforcing the need for adjustments to the instructions and kit components (Table 1).

**Table 1:** Bivariate analysis between sociodemographic characteristics and self-test usability, São Paulo, São Paulo, Brazil

Variables	High usability n (%)	Low usability n (%)	*p* value
**Sex**			
Male	13 (31.7%)	28 (68.3%)	0.3902
Female	20 (40.8%)	29 (59.2%)	
**Has children**			
Yes	22 (33.8%)	43 (66.2%)	0.465
No	11 (44.0%)	14 (56.0%)	
**Sexual orientation**			
Heterosexual	26 (40.6%)	38 (59.4%)	0.2404
Non-heterosexual	7 (26.9%)	19 (73.1%)	
**Age group**			
30 years or less	11 (45.8%)	13 (54.2%)	0.3263
More than 30 years	22 (33.3%)	44 (66.7%)	
**Years of schooling**			
12 years or less	22 (44.0%)	28 (56.0%)	0.1269
More than 12 years	11 (27.5%)	29 (72.5%)	
**Has formal income**			
Yes	16 (27.6%)	42 (72.4%)	0.002
No	17 (53.1%)	15 (46.9%)	
**Income**			
Below 2 minimum wages	2 (28.6%)	5 (71.4%)	1.0
2 minimum wages or more	31 (37.3%)	52 (62.7%)	

**Figure 1:**
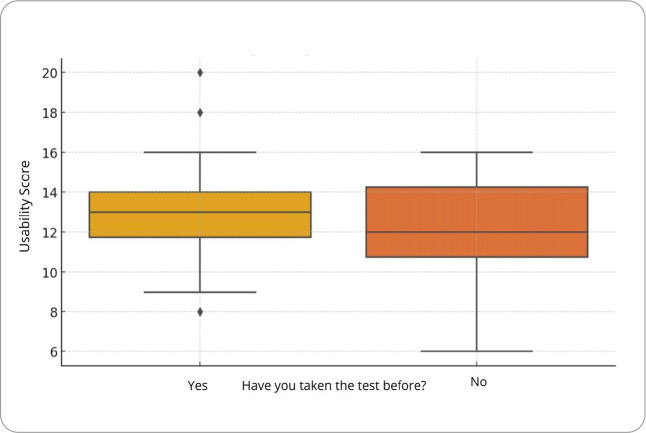
Usability score by previous experience with COVID-19 self-testing, São Paulo, São Paulo, Brazil

When comparing the overall usability score between participants who had previously taken a diagnostic test for COVID-19 (experienced group) and those without prior experience, a trend toward higher perceived usability was observed among those with previous experience. The mean score was 13.0 points in the experienced group and 12.0 points in the group without experience (Figure 1). Although the experienced group showed slightly higher and less dispersed values, no statistically significant difference was observed between the groups (p = 0.121).

Most participants (81.4%) reported difficulties when performing the COVID-19 self-test. Among these difficulties, the most frequent were understanding the instructions (70%), the use of the swab and buffer (45% each), understanding the procedure (40%), and reading the result (30%). These findings suggest limitations in the clarity of the instructions and in handling the self-test (Table 2).

**Table 2:** Frequency of self-reported difficulties in the use of the COVID-19 antigen self-test, São Paulo, São Paulo, Brazil

Variable	Category	n	%
Experienced difficulties	Yes	70	81.4
	No	16	18.6
Difficulty understanding the instructions in the guide	Yes	14	70.0
	No	6	30.0
Difficulty understanding the product leaflet	No	13	65.0
	Yes	7	35.0
Difficulty opening the pouch	No	13	65.0
	Yes	7	35.0
Difficulty using the nasal swab	No	11	55.0
	Yes	9	45.0
Difficulty using the dropper	No	17	85.0
	Yes	3	15.0
Difficulty opening and/or dripping the buffer	No	11	55.0
	Yes	9	45.0
Difficulty understanding how to perform the test	No	12	60.0
	Yes	8	40.0
Difficulty reading the result	No	14	70.0
	Yes	6	30.0
Other difficulties	No	14	70.0
	Yes	6	30.0

In the multivariable analysis, only income above two minimum wages showed a statistically significant association with high self-test usability (aOR = 1.92; 95% CI: 1.15-5.67; p = 0.009), indicating that participants with higher income were almost twice as likely to rate the self-test as easy to use (Table 3).

**Table 3:** Multivariable analysis of factors associated with high usability of the COVID-19 antigen self-test, São Paulo, São Paulo, Brazil

Variables	aOR	IC95%	*p* value
Sex	1.25	0.48 - 3.28	0.646
Children	1.88	0.57 - 6.14	0.297
Sexual orientation	0.69	0.21 - 2.19	0.524
Age	0.35	0.11 - 1.09	0.07
Years of schooling	0.48	0.17 - 1.37	0.171
Has formal income	2.46	0.87 - 6.98	0.09
Income above 2 minimum wages	1.92	1.15 - 5.67	0.009

In addition, it is important to highlight participants’ accounts from the interviews, which illustrate the main difficulties they faced when performing the self-test. These statements reveal practical and comprehension-related obstacles, reinforcing the limitations identified in the statistical findings.

Among the difficulties related to handling the nasal swab, Participant 19, for example, reported: “*Difficulty opening and breaking the swab*”, while Participant 26 mentioned uncertainty regarding the depth of insertion: “*Difficulty inserting it as far as necessary*”. In addition, Participant 62 noted: “*It was difficult to identify the 2.5 cm limit that needs to be inserted into the nose*”.

Problems understanding the instructions were also frequently reported. Participant 23 highlighted the legibility of the material: “Small print in the instruction guide”, and Participant 50 stated: “*I couldn’t see the letters to read*” In some cases, the content was not even read in its entirety, as reported by Participant 52: “*I didn’t have the patience to read the quick guide*”.

Finally, there were also difficulties involving the use of other test components and the interpretation of results. Participant 59 reported multiple obstacles: “*I couldn’t see properly*” (referring to reading the leaflet and the results) and “*I couldn’t open the buffer cap*”.

These accounts reinforce that the main challenges involve the clarity of the instructions, the legibility of the materials, and the technical handling of the self-test components. The participants’ statements underscore the need for adaptations in the design and communication of self-tests in order to promote accessibility and autonomy among this group of users.

## DISCUSSION

Our findings indicate that, although the COVID-19 antigen self-test has the potential to be an accessible tool for home-based diagnosis, its usability still faces important limitations for some users, given that only about one-third of participants classified the test as having high usability, based on barriers related both to understanding and to the practical execution of the procedure.

However, international studies on the usability of COVID-19 self-tests report success rates for autonomous use that are generally higher than those observed in the present study. For example, Bresser^(28)^, evaluating the use of self-tests in Lesotho and Zambia, found that approximately 70% of participants were able to correctly perform all test steps without supervision, demonstrating high overall usability. Similarly, Mukoka^(22)^, in an assessment conducted in Malawi, reported that more than 80% of users correctly completed the self-test, even in populations with low educational attainment.

These discrepancies should be interpreted in light of differences in the context of implementation, the instructional materials provided, the type and model of self-test used, as well as the support received by users during the process. For instance, in both cited studies, participants received simplified visual instructions and, in some cases, explanatory videos or supervised support, which may have facilitated correct execution of the procedure compared to our study, in which this did not occur.

In addition, the degree of prior familiarity with self-care technologies and the average level of health literacy in the populations assessed also appear to directly influence perceived usability. A systematic review on COVID-19 self-tests showed that the combination of accessible educational materials, community-based technical support, and intuitive product design is strongly associated with greater acceptance and lower error rates during home use^(29)^. This scenario suggests that the mere provision of a self-test does not translate into its correct or efficient use.

In Brazil, although self-tests have gained widespread uptake in recent years, especially for COVID-19 and HIV, systematic studies on their usability remain scarce^(7,12,25)^. Since authorization in 2022, ANVISA has received 33 registration requests for COVID-19 self-tests, with priority review while the health emergency was in effect^(4)^. In addition, initiatives such as the expansion of HIV self-testing among key populations have helped consolidate this technology as a central testing strategy in the country.

Nevertheless, despite this broad distribution, the national scientific literature still lacks robust and systematic evaluations of how these devices are used in practice, whether users understand the instructions, are able to correctly perform the procedure, and interpret the results safely. For example, a study^(30)^ conducted in 2022 reported accuracy rates above 60% for performing HIV self-tests in supervised settings, with a significant reduction when the tests were performed in home settings without direct professional support, reinforcing that the simple availability of the test does not guarantee its effective use.

Thus, national and international literature supports our findings, suggesting that variables such as education, income, familiarity with health technologies, and levels of health literacy can significantly affect the user experience. Reinforcing this result, the multivariable analysis in our study showed that an income above two minimum wages was the only factor statistically associated with a higher likelihood of rating the self-test as highly usable.

It is also important to highlight that approximately 81.4% of participants reported having experienced at least one difficulty when using the self-test, with the most common related to understanding the instructions (70%). This finding shows that the user experience can be substantially compromised when instructional materials are not sufficiently clear, accessible, and adapted to the users’ sociocultural profile. The absence of technical support, combined with complex or minimally visual instructions, tends to exacerbate uncertainty during the execution of the procedure and interpretation of results, especially among individuals with lower health literacy.

These findings are consistent with other studies, which point to the complexity of certain self-test components as a limiting factor for their widespread use^(31)^. International research on HIV self-tests^(32)^ indicates that difficulty understanding the instructions in the leaflet and package insert is common and suggests that the support materials provided with the self-test may not be culturally sensitive or adequately adapted, and therefore may be inadequate for the needs of the target population. Lengthy instructions, technical language, and the absence of clear visual elements can hinder comprehension, especially for people with lower levels of health literacy. Strategies such as the use of simplified language, infographics, explanatory videos, and remote support could help mitigate this problem.

In the present study, qualitative reports and difficulties related to the physical handling of components such as the swab and buffer (45%) reinforce how the design of self-tests, which typically includes a nasal swab, buffer, and dropper, can represent an important barrier. These steps require a certain degree of motor coordination and confidence to perform correctly, which can be challenging for individuals with lower manual dexterity or who feel insecure about the procedure. Simplifying the design of self-test kits is a frequent recommendation in the literature to improve user experience and reduce errors^(33)^.

Another important issue is the difficulty interpreting results (30%), a critical aspect for the self-test to fulfill its role in informing and guiding user behavior. The presence of faint or barely visible lines on the devices can generate doubts, especially in the absence of a health professional. Studies suggest that uncertainty in reading the result may lead both to a false sense of security and to unnecessary anxiety^(34)^, making the self-testing experience unpleasant for the user.

Finally, the results of this study point to the urgent need to incorporate an equity lens into the design and implementation of health technologies, especially those aimed at self-administered diagnosis. The low perceived usability identified among participants undermines the potential of self-tests as an effective public health strategy, particularly in contexts of greater social vulnerability and limited access to formal diagnostic services.

Although self-tests represent an advance in addressing infections such as COVID-19, their effectiveness depends directly on users’ ability to correctly understand, perform, and interpret the procedure. This requires more than technical quality in product development; it demands an institutional commitment to educational actions, culturally appropriate instructional materials, and ongoing community support.

In this sense, it is essential that public policies aimed at expanding rapid testing go beyond the mere distribution of self-tests and be accompanied by support strategies that promote real accessibility, confidence in use, and user autonomy. In the context of the Brazilian Unified Health System (SUS), grounded in the principles of universality, comprehensiveness, and equity, it becomes crucial to avoid technological polarization, which tends to benefit only groups with higher levels of education or greater informational and digital capital.

Popularizing diagnostic technologies means bringing them closer to the concrete realities of the Brazilian population, considering varying levels of literacy, education, language, culture, housing conditions, and access to information. This involves investing in easy-to-understand visual materials, user-centered device design, and local initiatives involving community health workers, thereby strengthening the bridge between innovation and social justice.

In this way, self-tests will be able to truly fulfill their role as instruments of care, contributing to epidemiological surveillance, self-care, and equitable access to diagnostic services.

### Study limitations

The study has some limitations that should be considered when interpreting the results. First, the small sample size may limit the generalizability of the findings to other populations. However, the sample allows for a robust exploratory analysis of the perceived usability of the self-test and its possible associations with sociodemographic variables, providing relevant input for future research and for improving testing strategies among vulnerable populations.

In addition, the study was conducted in a specific context, which may not reflect the difficulties and perceptions of other population groups in different regions or with distinct socioeconomic characteristics. Finally, it was not possible to control for variables such as prior experience with self-tests or other diagnostic devices, which may have influenced the ease of use reported by participants.

### Contributions to the field

Our study highlights the importance of incorporating the perspective of social inequities into the design and implementation of strategies involving diagnostic self-tests by demonstrating that socioeconomic determinants, such as income, influence the perception of usability. By identifying barriers related to understanding instructions and handling diagnostic supplies, the study provides input for improving instructional materials and optimizing usability-oriented kit design, with a focus on accessibility and safety for socially vulnerable populations.

Additionally, the findings have transdisciplinary applicability and may be extended to the development of more inclusive guidance for other health technologies used in the home setting, such as self-administered treatment regimens, remote monitoring devices, and self-care strategies. In this way, the study contributes to strengthening user-centered care practices, improving supervised self-care, and promoting equity in access to and adherence to therapeutic interventions in out-of-hospital settings.

## CONCLUSIONS

Despite the promising potential of COVID-19 antigen self-tests as a tool for expanding large-scale diagnostic coverage, the findings of this study reveal a somewhat more complex reality: perceived usability is generally low. Only one-third of participants considered the test easy to use, while most reported difficulties at various stages of the process, from reading the instructions to interpreting the result.

Furthermore, among the factors investigated, only higher income was significantly associated with high usability. This association indicates that, more than a technical issue, the ability to use a self-test autonomously may reflect structural inequalities. Finally, we believe there is an urgent need to rethink the design and implementation of self-tests, as simply distributing them is not enough. It is necessary to ensure that instructions are accessible in terms of language, format, and context; that educational support is available within communities; and that the development of health technologies incorporates the principle of equity from the outset.

## Data Availability

The research data are available only upon request.
