# Supplementary material for: Usabilidade de autoteste de antígeno para COVID-19: fatores associados e desafios relatados
Source: Rev Bras Enferm. 2026 Aug 3;79:e20250197. doi: 10.1590/0034-7167-2025-0197 (PMC13432805; doi:10.1590/0034-7167-2025-0197)
Supplement: Translation

# Translation: Usabilidade de autoteste de antígeno para COVID-19: fatores associados e desafios relatados

**DOI:** 10.1590/0034-7167-2025-0197pt

**Authors:** Isadora Silva de Carvalho, Lariane Angel Cepas, Talita Morais Fernandes, Carlos Arterio Sorgi, Ana Flávia Nacif Pinto Coelho Pires, Álvaro Francisco Lopes de Sousa, Isabella Silva de Carvalho, Ana Paula Morais Fernandes

**Affiliations:** I Universidade de São Paulo, Escola de Enfermagem de Ribeirão Preto. Ribeirão Preto, São Paulo, Brasil; II Instituto Superior de Lisboa e Vale do Tejo. Lisboa, Lisboa, Portugal; III Faculdade de Filosofia Ciências e Letras. Ribeirão Preto, São Paulo, Brasil; IV Universidade de Brasília. Brasília, Distrito Federal, Brasil; V Universidade Federal do Mato Grosso do Sul. Três Lagoas, Mato Grosso do Sul, Brasil; VI Escola Nacional de Saúde Pública. Lisboa, Lisboa, Portugal; VII Universidade Estadual Paulista “Júlio de Mesquita Filho”. Jaboticabal, São Paulo, Brasil

**Keywords:** Autoteste, COVID-19, Testes de Diagnóstico Rápido, Acessibilidade aos Serviços de Saúde, Populações Vulneráveis.

## Abstract

**Objetivos:**

avaliar a usabilidade de um autoteste de antígeno para COVID-19 e identificar fatores associados à percepção de facilidade de uso.

**Métodos:**

estudo transversal realizado em São Paulo (SP), Brasil, entre junho e julho de 2023, com 90 participantes que utilizaram o autoteste e responderam a um questionário estruturado. Foram feitas análises descritivas, bivariadas e de regressão logística multivariada.

**Resultados:**

apenas 33 participantes (36,7%) relataram alta usabilidade. Ter renda acima de dois salários mínimos foi significativamente associado à maior chance de avaliar o teste como fácil de usar (ORa = 1,92; IC95%: 1,15-5,67; P= 0,009). As principais dificuldades envolveram a compreensão das instruções (70%) e o manuseio de componentes como *swab* e tampão (45%).

**Conclusões:**

embora promissor para ampliar o acesso ao diagnóstico, o autoteste enfrenta barreiras ligadas à compreensão e ao manuseio, especialmente entre pessoas de menor renda. Esses achados reforçam a importância de instruções claras e adaptações tecnológicas conforme o perfil dos usuários.

## INTRODUÇÃO

A pandemia de *Coronavirus Disease* 2019 (COVID-19), declarada em 2020, teve impacto profundo e duradouro sobre a saúde pública global. Até o encerramento das principais emergências internacionais, em 2023, o mundo havia registrado mais de 700 milhões de casos confirmados e aproximadamente 7 milhões de mortes atribuídas à doença^(1)^. O Brasil esteve entre os países mais afetados, com mais de 37 milhões de casos e mais de 700 mil óbitos, refletindo não apenas a virulência do SARS-CoV-2, mas também as fragilidades estruturais do sistema de saúde e a desigualdade social^(2)^.

No contexto brasileiro, diversas estratégias foram implementadas para conter a disseminação da COVID-19, tais como campanhas de conscientização sobre a higienização frequente das mãos, distanciamento social, uso de máscaras em locais públicos e campanhas de vacinação em larga escala. No entanto, esta última estratégia se consolidou como a principal medida de prevenção somente a partir de 2021. No período inicial da pandemia, quando ainda não havia imunizantes disponíveis para toda a população, a testagem em massa de indivíduos sintomáticos ou expostos desempenhou papel fundamental no controle da transmissão. A realização de testes diagnósticos permitiu a identificação rápida de casos, o isolamento precoce de pessoas infectadas e o rastreamento de contatos, estratégias essenciais para mitigar o avanço da doença, principalmente em um cenário de alta transmissibilidade e circulação comunitária do vírus.

Nesse sentido, entre as ações adotadas, a ampliação do acesso aos testes rápidos de antígeno se destacou por possibilitar o diagnóstico rápido, com menor custo e sem necessidade de infraestrutura laboratorial complexa^(3)^. Posteriormente, a introdução dos autotestes, autorizada no Brasil pela Agência Nacional de Vigilância Sanitária (ANVISA) em 2022, representou um avanço importante ao permitir que os próprios usuários realizassem o teste em casa, de maneira prática, sigilosa e autônoma^(4)^. Entende-se que essa medida ampliou o acesso ao diagnóstico, especialmente entre populações com dificuldades de deslocamento ou que classicamente enfrentavam barreiras de acesso aos serviços de saúde^(5,6)^.

Entretanto, apesar do avanço representado pela autorização dos autotestes, sua adoção em larga escala no Brasil ainda enfrenta desafios relevantes^(7)^. Diferentemente dos testes realizados em ambiente laboratorial ou sob supervisão de profissionais de saúde, o uso do autoteste pressupõe que o próprio indivíduo seja capaz de compreender as instruções fornecidas, executar corretamente todas as etapas do procedimento e interpretar adequadamente o resultado obtido^(8,9)^. Essa autonomia, embora potencialize o acesso ao diagnóstico, também impõe barreiras adicionais, sobretudo para grupos populacionais com menor nível de escolaridade, baixa literacia em saúde ou acesso restrito a informações de qualidade.

Experiências prévias com outros tipos de autotestes, como o de diagnóstico para a infecção pelo HIV e o de glicemia capilar para monitoramento do diabetes, demonstram que dificuldades relacionadas ao entendimento das orientações, à execução técnica dos procedimentos e à interpretação dos resultados não são exclusivas do contexto da COVID-19^(8,10)^. Estudos sobre autoteste de HIV no Brasil, por exemplo, relatam que participantes frequentemente têm dúvidas sobre o uso do kit, temor de cometer erros durante o procedimento e insegurança diante de resultados positivos, especialmente na ausência de orientação profissional ou material educativo acessível^(11,12)^. De modo semelhante, usuários de glicosímetros relatam obstáculos quanto ao manuseio do equipamento, à correta coleta da amostra de sangue e ao entendimento dos valores aferidos, sobretudo entre idosos ou pessoas com baixa escolaridade^(13)^.

Essas evidências sugerem que, embora os autotestes ampliem a autonomia e o alcance das estratégias de diagnóstico, sua efetividade depende diretamente do suporte oferecido aos usuários, da clareza das instruções e da adequação dos materiais ao perfil sociocultural da população. Dificuldades persistentes podem comprometer tanto a precisão dos resultados quanto a confiança e a adesão à testagem domiciliar, reiterando a importância de políticas públicas e de materiais instrucionais adaptados às realidades socioculturais brasileiras^(13)^.

Esse cenário se mostrou especialmente desafiador para diversos grupos populacionais historicamente vulnerabilizados, como pessoas lésbicas, gays, bissexuais, transgêneros, queer, intersexuais, assexuais, pansexuais e outras identidades (LGBTQIAP+) e trabalhadores do sexo, que já enfrentam barreiras estruturais no acesso à educação, saúde e trabalho decente, conforme os Objetivos de Desenvolvimento Sustentável (ODS), especialmente de número 8, marcadas por estigma, discriminação e insegurança econômica^(14)^. Para esses grupos, a adoção do autoteste, apesar de promissora, pode ser ainda mais complexa diante das limitações de acesso a informações claras, suporte técnico e material instrucional adaptado à sua realidade^(15,16)^.

Durante os anos mais críticos da pandemia, essas populações estavam mais expostas ao risco de infecção, muitas vezes por atuarem em atividades informais e não terem condições de adotar medidas eficazes de distanciamento^(17)^. Além disso, a disseminação de desinformação e a baixa literacia em saúde dificultaram a adesão às orientações sanitárias e à correta utilização de dispositivos diagnósticos, como os autotestes^(18-20)^.

Diversos estudos destacaram a importância dos autotestes como ferramentas complementares aos sistemas formais de diagnóstico^(21,22)^. Contudo, também apontaram limitações relacionadas à compreensão das instruções, à complexidade de manuseio dos materiais e à leitura de resultados, especialmente entre indivíduos com baixa escolaridade ou acesso limitado à informação^(23,24)^.

Considerando o crescente interesse na ampliação do uso de autotestes para diversas condições de saúde, torna-se essencial compreender as barreiras enfrentadas pelos usuários, a fim de garantir não apenas a eficácia desses dispositivos, mas também sua aceitação e adesão ao seu uso. No entanto, uma revisão sistematizada da literatura revela a escassez de estudos nacionais que avaliem a usabilidade dos autotestes em contextos reais, especialmente entre populações vulneráveis. A análise dessas experiências pode gerar evidências relevantes para o aprimoramento das estratégias de implementação, além de contribuir para a expansão do uso efetivo dos autotestes no diagnóstico de outras condições de saúde, para além da COVID-19.

## OBJETIVOS

Avaliar a usabilidade de um autoteste de antígeno para COVID-19 e identificar fatores associados à percepção de facilidade de uso.

## MÉTODOS

### Aspectos Éticos

O estudo foi aprovado pelo Comitê de Ética em Pesquisa da Faculdade de Filosofia, Ciências e Letras de Ribeirão Preto (CAAE: 51994921.7.0000.5407), e todos os participantes assinaram o termo de consentimento livre e esclarecido por escrito.

### Tipo de Estudo

Trata-se de um estudo transversal, de abordagem analítica, cujo delineamento e descrição seguem as recomendações da lista de verificação *Strengthening the Reporting of Observational Studies in Epidemiology* (STROBE).

### Local do Estudo

A coleta de dados foi realizada no município de São Paulo (SP), entre junho e julho de 2023, período marcado pela consolidação da vacinação contra a COVID-19 no Brasil e pela ampliação das políticas de testagem rápida na população. A pesquisa ocorreu no contexto da ação “*USP na Comunidade*”, promovida pela Pró-Reitoria de Cultura e Extensão Universitária da Universidade de São Paulo (USP), em parceria com o Programa Conjunto das Nações Unidas sobre HIV/AIDS no Brasil (UNAIDS). Essa iniciativa, voltada à promoção da saúde e da cidadania, utiliza Unidades Móveis para oferecer serviços, atividades educativas e orientação à população em espaços públicos de grande circulação.

O Memorial da América Latina foi escolhido como local para a realização da pesquisa devido à sua localização estratégica, ampla circulação de públicos diversos e consolidada tradição em sediar ações de educação, cidadania e saúde pública. Situado em uma região de fácil acesso na cidade de São Paulo, o Memorial recebe diariamente um público heterogêneo, incluindo estudantes, trabalhadores, famílias e grupos em situação de vulnerabilidade social. Essa diversidade de perfis contribuiu para garantir uma amostra variada e representativa, essencial para a avaliação da usabilidade dos autotestes em diferentes segmentos populacionais. Ademais, o reconhecimento do Memorial como espaço acolhedor e confiável favoreceu o engajamento dos participantes e a integração da pesquisa às campanhas educativas e de promoção da saúde conduzidas durante o evento.

### População e Amostra

A amostragem do estudo foi do tipo não probabilística, combinando conveniência e a estratégia de bola de neve, na qual os participantes iniciais eram convidados diretamente no local do estudo e, posteriormente, podiam indicar outras pessoas potencialmente elegíveis e interessadas em participar e testarem-se no local^(25)^. Foram incluídos indivíduos com idade igual ou superior a 18 anos, residentes no Brasil e com capacidade visível de compreensão e comunicação adequadas para responder à entrevista presencial. Os critérios de exclusão envolveram recusa em participar ou incapacidade de completar o questionário.

A amostra final foi composta por 90 participantes, número definido pela dinâmica de recrutamento durante o período do estudo. Apesar da natureza de conveniência, foi realizada uma análise de poder estatístico a posteriori para justificar a adequação da amostra. Para isso, consideraram-se um nível de significância (*alfa*) de 5%, poder estatístico (1*-β*) de 80%, *odds ratio* mínimo esperado de 2,5 para associação entre fatores sociodemográficos e alta usabilidade do teste, e uma prevalência de alta usabilidade estimada em aproximadamente 30% dos participantes. Nessas condições, a amostra de 90 indivíduos mostrou-se suficiente para detectar associações de magnitude moderada com razoável confiança estatística, conforme cálculo realizado no software G*Power versão 3.1.

### Protocolo do Estudo

A coleta de dados foi realizada por meio de entrevistas presenciais individuais, conduzidas por profissionais previamente treinados. O principal desfecho do estudo foi a “usabilidade do autoteste de antígeno para COVID-19”, definida como o grau em que um usuário, em condições reais, consegue compreender as instruções, executar corretamente o procedimento e interpretar o resultado de forma autônoma, segura e satisfatória. Esse conceito foi fundamentado na literatura internacional e adaptado ao contexto do presente estudo, considerando as recomendações da Resolução da Diretoria Colegiada (RDC) nº 595/2022 da ANVISA^(26)^ e protocolos validados por Bien-Gund^(27)^.

Para a coleta de dados, foi utilizado um questionário estruturado, desenvolvido com base em diretrizes da ANVISA (RDC nº 595/2022) e em instrumentos internacionais de avaliação de usabilidade. O questionário foi composto por quatro seções:

1. Características sociodemográficas - incluindo idade, identidade de gênero, orientação sexual, escolaridade, ocupação, renda familiar mensal, filhos, local de nascimento e tempo de residência na cidade/estado.
2. Conhecimento prévio sobre COVID-19 e autotestes: abordando, por exemplo, se o participante já havia realizado algum destes testes anteriormente.
3. Usabilidade do autoteste: contemplando itens relacionados à compreensão das instruções, manuseio dos componentes (*swab*, tampão, conta-gotas), execução do procedimento e leitura do resultado. As respostas foram predominantemente dicotômicas (sim/não), e a seção incluía também um campo aberto para que os participantes descrevessem, com suas próprias palavras, quaisquer dificuldades específicas encontradas durante a testagem.
4. Satisfação geral com o autoteste: avaliando a utilidade percebida das instruções, sugestões de melhoria e disposição para recomendar o teste a outras pessoas.

A seção de usabilidade do instrumento foi previamente testada por meio de um pré-teste com um grupo piloto de cinco participantes da população-alvo, para assegurar clareza, pertinência e adequação das questões ao conceito de usabilidade.

O estudo adotou uma abordagem quantitativa, utilizando um questionário estruturado composto por 22 perguntas fechadas, das quais 21 apresentavam formato binário (sim/não), direcionadas à identificação de barreiras práticas e informacionais no processo de autotestagem. Nos casos em que os participantes indicavam ter enfrentado alguma dificuldade, era disponibilizado um campo adicional para a descrição do tipo de dificuldade relatada, cujo conteúdo foi utilizado apenas para fins descritivos, sem análise qualitativa sistemática.

Para quantificar a usabilidade, foi criado um escore composto por 21 variáveis binárias referentes à ausência de dificuldades (resposta “não”) em diferentes etapas do autoteste, atribuindo-se 1 ponto para cada resposta indicativa de facilidade. Adicionalmente, foi incluída a nota de praticidade atribuída pelo participante, variando de 1 (nada prático) a 5 (muito prático), resultando em um escore total de 0 a 26 pontos, em que valores mais altos indicavam maior percepção de usabilidade. O ponto de corte para classificação em “alta usabilidade” *versus* “média ou baixa usabilidade” foi definido com base na mediana da amostra, estratégia frequentemente empregada em estudos exploratórios quando não há pontos de corte validados na literatura. A consistência interna do escore foi avaliada por meio do alfa de Cronbach (valor obtido: 0,81), indicando boa confiabilidade interna do construto.

### Análise estatística

As análises estatísticas foram conduzidas utilizando o *software R*, versão 4.1.3. Inicialmente, foi realizada uma análise descritiva univariada, com cálculo de frequências absolutas e relativas para variáveis categóricas e medidas de tendência central e dispersão para variáveis contínuas. Valores ausentes ou omissos foram identificados e tratados como dados faltantes, sem substituição.

Para a análise bivariada, foram utilizados testes de associação entre a variável dependente, usabilidade do autoteste (classificada como variável binária: 1 = alta usabilidade; 0 = média ou baixa), e variáveis independentes de natureza sociodemográfica. As associações foram avaliadas por meio do teste do qui-quadrado de Pearson ou, quando necessário, do teste exato de Fisher (p < 0,05).

As variáveis independentes incluíram variáveis sociais e demográficas. Todas as variáveis foram previamente dicotomizadas para fins de análise multivariada. A seguir, foi ajustado um modelo de regressão logística binária com o desfecho “alta usabilidade do teste”. As variáveis com p < 0,20 na análise bivariada foram incluídas no modelo multivariado. Os resultados foram expressos em *odds ratios* ajustados (ORa) com IC95% e valores de p. A qualidade do ajuste do modelo foi avaliada por meio do teste da razão de verossimilhança (p < 0,01), bem como pelos coeficientes de pseudo R^2^ de Cox-Snell (0,27), Nagelkerke (0,49) e McFadden (0,39).

Além disso, a curva ROC (*Receiver Operating Characteristic*) foi utilizada para avaliar o poder discriminativo do modelo final, resultando em uma área sob a curva (AUC) de 0,86, indicando uma forte capacidade preditiva.

## RESULTADOS

Participaram 90 sujeitos. A média do escore de usabilidade foi 12,0 pontos (em um máximo teórico de 26), sugerindo que, de forma geral, os usuários avaliaram o autoteste como moderadamente usável. A mediana de 13 indica que metade dos participantes atingiu esse nível de usabilidade, o que se alinha à classificação de usabilidade média. Considerando o ponto de corte adotado (mediana do escore), 33 participantes (36,7%; IC95%: 26,6%-47,8%) foram classificados como apresentando alta usabilidade do autoteste de antígeno para COVID-19.

Cerca de um terço dos participantes relatou alta usabilidade, enquanto uma parcela menor experimentou baixa usabilidade. A presença de participantes com escore mínimo (0) mostra que ainda há desafios pontuais de entendimento e execução do autoteste, reforçando a necessidade de ajustes nas instruções e componentes do kit (Tabela 1).

**Tabela 1:** Análise bivariada entre características sociodemográficas e usabilidade do autoteste, São Paulo, São Paulo, Brasil

Variáveis	Alta Usabilidade	Baixa Usabilidade	Valor de *p*
**Sexo**			
Masculino	13 (31,7%)	28 (68,3%)	0,3902
Feminino	20 (40,8%)	29 (59,2%)	
**Possui filhos**			
Sim	22 (33,8%)	43 (66,2%)	0,465
Não	11 (44,0%)	14 (56,0%)	
**Orientação sexual**			
Heterossexual	26 (40,6%)	38 (59,4%)	0,2404
Não-Heterossexual	7 (26,9%)	19 (73,1%)	
**Faixa etária**			
30 anos ou menos	11 (45,8%)	13 (54,2%)	0,3263
Mais de 30 anos	22 (33,3%)	44 (66,7%)	
**Anos de estudo**			
12 anos ou menos	22 (44,0%)	28 (56,0%)	0,1269
Mais de 12 anos	11 (27,5%)	29 (72,5%)	
**Possui Renda Formal**			
Sim	16 (27,6%)	42 (72,4%)	0,002
Não	17 (53,1%)	15 (46,9%)	
**Renda**			
Abaixo de 2 salários mínimo	2 (28,6%)	5 (71,4%)	1,0
2 salário mínimos ou mais	31 (37,3%)	52 (62,7%)	

Ao comparar o escore geral de usabilidade entre participantes que já haviam realizado um teste diagnóstico de COVID-19 anteriormente (grupo experiente) e aqueles sem experiência prévia, observou-se uma tendência de maior usabilidade percebida entre os mais experientes. A média do escore foi de 13,0 pontos no grupo com experiência e 12,0 pontos no grupo sem experiência (Figura 1). Embora o grupo experiente tenha apresentado valores ligeiramente mais altos e menos dispersos, não se observou diferença estatisticamente significativa entre os grupos (p = 0,121).

**Figura 1:** Escore de usabilidade por experiência prévia com autoteste para COVID-19, São Paulo, São Paulo, Brasil

A maioria dos participantes (81,4%) relatou dificuldades ao realizar o autoteste de COVID-19. Dentre as dificuldades, destacam-se a compreensão das instruções (70%), o uso do *swab* e do tampão (45% cada), o entendimento do procedimento (40%) e a leitura do resultado (30%). Esses achados sugerem limitações na clareza das orientações e no manuseio do autoteste (Tabela 2).

**Tabela 2:** Frequência de dificuldades autorreferidas no uso do autoteste de antígeno para COVID-19, São Paulo, São Paulo, Brasil

Variável	Categoria	n	%
Teve dificuldades	Sim	70	81,4
	Não	16	18,6
Dificuldade de entender as instruções do guia	Sim	14	70,0
	Não	6	30,0
Dificuldade de entender a bula do produto	Não	13	65,0
	Sim	7	35,0
Dificuldade na abertura da bolsa	Não	13	65,0
	Sim	7	35,0
Dificuldade com o uso do *swab* de coleta nasal	Não	11	55,0
	Sim	9	45,0
Dificuldade para usar o conta-gotas	Não	17	85,0
	Sim	3	15,0
Dificuldade para abrir e/ou pingar o tampão	Não	11	55,0
	Sim	9	45,0
Dificuldade de entender como fazer o teste	Não	12	60,0
	Sim	8	40,0
Dificuldade para ler o resultado	Não	14	70,0
	Sim	6	30,0
Outras dificuldades	Não	14	70,0
	Sim	6	30,0

Na análise multivariada, apenas a renda superior a dois salários mínimos apresentou associação estatisticamente significativa com a alta usabilidade do autoteste (ORa = 1,92; IC95%: 1,15-5,67; p = 0,009), indicando que participantes com maior renda tiveram quase o dobro de chance de avaliar o autoteste como fácil de usar (Tabela 3).

**Tabela 3:** Análise multivariada dos fatores associados à alta usabilidade do autoteste de antígeno para COVID-19, São Paulo, São Paulo, Brasil

Variáveis	ORa	IC95%	Valor de *p*
Sexo	1,25	0,48 - 3,28	0,646
Filhos	1,88	0,57 - 6,14	0,297
Orientação sexual	0,69	0,21 - 2,19	0,524
Idade	0,35	0,11 - 1,09	0,07
Anos de estudo	0,48	0,17 - 1,37	0,171
Possui renda formal	2,46	0,87 - 6,98	0,09
Renda acima de 2 salários mínimo	1,92	1,15 - 5,67	0,009

Além disso, é importante destacar os relatos dos participantes durante as entrevistas que ilustram as principais dificuldades enfrentadas ao realizar o autoteste. Os depoimentos evidenciam obstáculos práticos e de compreensão, reforçando as limitações identificadas nos achados estatísticos.

Entre as dificuldades relacionadas ao manuseio do *swab* nasal, o Participante 19, por exemplo, relatou: *“Dificuldade para abrir o swab e quebrar”*, enquanto o Participante 26 mencionou insegurança quanto à profundidade da inserção: *“Dificuldade para introduzir até onde é necessário”*. Além disso, o Participante 62 apontou: *“Difícil identificar o limite de 2,5 cm que precisa ser introduzido no nariz”*.

Também foram relatados com frequência problemas de compreensão das instruções. O Participante 23 destacou a legibilidade do material: *“Letra pequena no guia de instruções”*, e o Participante 50 afirmou: *“Não enxerguei as letras para ler”*. Em alguns casos, o conteúdo sequer foi lido por completo, como relatado pelo Participante 52: *“Não tive paciência para ler o guia rápido”*.

Por fim, houve ainda dificuldades envolvendo o uso de outros componentes do teste e a interpretação dos resultados. O Participante 59 relatou múltiplos obstáculos: *“Não enxerguei direito”* (referindo-se à leitura da bula e dos resultados) e *“Não consegui abrir o tampão”*.

Esses depoimentos reforçam que os principais desafios envolvem a clareza das instruções, a legibilidade dos materiais e o manuseio técnico dos itens do autoteste. As falas evidenciam a necessidade de adaptações no design e na comunicação dos autotestes, a fim de promover acessibilidade e autonomia entre este grupo de usuários.

## DISCUSSÃO

Nossos achados indicam que, embora o autoteste de antígeno para COVID-19 tenha potencial de ser uma ferramenta acessível para diagnóstico domiciliar, sua usabilidade ainda encontra limitações importantes entre alguns usuários, visto que apenas cerca de um terço dos participantes classificou o teste como de alta usabilidade com base em barreiras tanto de compreensão quanto de execução prática do procedimento.

No entanto, estudos internacionais sobre usabilidade de autotestes para COVID-19 apontam prevalências de sucesso no uso autônomo, geralmente superiores às observadas no presente estudo. Por exemplo, Bresser^(28)^, avaliando a utilização de autotestes em Lesoto e Zâmbia, encontraram que aproximadamente 70% dos participantes foram capazes de realizar corretamente todas as etapas do teste sem supervisão, demonstrando alta usabilidade geral. De forma semelhante, Mukoka^(22)^, em avaliação realizada no Malaui, relataram que mais de 80% dos usuários completaram corretamente o autoteste, mesmo em populações de baixa escolaridade.

A existência dessas discrepâncias deve ser vista à luz das diferenças no contexto da aplicação, nos materiais instrucionais fornecidos, no tipo e modelo de autoteste utilizado, bem como no suporte recebido pelos usuários durante o processo. Por exemplo, em ambos os estudos citados, os participantes receberam instruções visuais simplificadas e, em alguns casos, vídeos explicativos ou suporte supervisionado, o que pode ter favorecido a execução correta do procedimento em comparação ao nosso estudo, em que isso não aconteceu.

Além disso, o grau de familiaridade prévia com tecnologias de autocuidado e o nível médio de literacia em saúde das populações avaliadas também parecem influenciar diretamente a usabilidade percebida. Estudo de revisão sistemática sobre autotestes de COVID-19 mostrou que a combinação entre materiais educativos acessíveis, apoio técnico comunitário e desenho intuitivo do produto está fortemente associada à maior aceitação e menor taxa de erros durante o uso domiciliar^(29)^. Esse cenário aponta que o simples fornecimento de autoteste não se traduz no seu uso correto ou eficiente.

No Brasil, embora os autotestes tenham ganhado ampla adesão nos últimos anos, especialmente para COVID-19 e HIV, estudos sistemáticos sobre sua usabilidade permanecem escassos^(7,12,25)^. Desde a autorização em 2022, a ANVISA registrou 33 pedidos de registro para autotestes de COVID-19, com análise prioritária enquanto vigorou a emergência sanitária^(4)^. Além disso, iniciativas como a ampliação do uso do autoteste de HIV por populações-chave têm contribuído para consolidar essa tecnologia como uma estratégia central de testagem no país.

No entanto, apesar dessa ampla distribuição, a literatura científica nacional ainda carece de avaliações robustas e sistemáticas sobre como esses dispositivos são utilizados na prática, se os usuários compreendem as instruções, conseguem realizar o procedimento corretamente e interpretam os resultados de forma segura. A exemplo disso, um estudo^(30)^ realizado em 2022 apontou taxas de acerto acima de 60% na execução do autoteste de HIV em ambientes supervisionados, com redução significativa quando feito em contextos domiciliares e sem apoio profissional direto, reforçando que a simples disponibilidade do teste não garante seu uso eficaz.

Dessa forma, a literatura nacional e internacional corrobora nossos achados, sugerindo que variáveis como escolaridade, renda e familiaridade com tecnologias em saúde e literacia em saúde podem comprometer significativamente a experiência de uso. Reforçando este achado, a análise multivariada em nosso estudo revelou que a renda superior a dois salários mínimos foi o único fator associado de forma estatisticamente significativa à maior chance de avaliar o autoteste como altamente usável.

Ainda é relevante destacar que cerca de 81,4% dos participantes relataram ter enfrentado pelo menos uma dificuldade ao utilizar o autoteste, sendo a principal delas relacionada à compreensão das instruções (70%). Esse dado evidencia que a experiência de uso pode ser substancialmente comprometida quando os materiais instrucionais não são suficientemente claros, acessíveis e adaptados ao perfil sociocultural dos usuários. A ausência de suporte técnico, aliada a instruções complexas ou pouco visuais, tende a acentuar inseguranças durante a execução do procedimento e a interpretação dos resultados, especialmente entre indivíduos com menor literacia em saúde.

Esses achados são consistentes com outros estudos, que apontam a complexidade de alguns componentes dos autotestes como um fator limitante para seu uso generalizado^(31)^. Pesquisas internacionais com autotestes para HIV^(32)^ apontam que a dificuldade em entender as instruções do guia e da bula é frequente e indicam que o material de apoio fornecido com o autoteste pode não ser culturalmente sensível ou adaptado, sendo, dessa forma, inadequado às necessidades do público-alvo. Instruções extensas, linguagem técnica e ausência de elementos visuais claros podem dificultar a compreensão, especialmente para pessoas com menor nível de literacia em saúde. Estratégias como o uso de linguagem simplificada, infográficos, vídeos explicativos e suporte remoto poderiam contribuir para minimizar esse problema.

No presente estudo, os relatos qualitativos e as dificuldades relacionadas com o manuseio físico de componentes, como o *swab* e o tampão (45%), reforçam como o design dos autotestes, composto por *swab* nasal, tampão e conta-gotas, pode representar uma barreira importante. Essas etapas exigem certo grau de coordenação motora e segurança para a execução correta, o que pode ser desafiador para pessoas com menor habilidade manual ou inseguras em relação ao procedimento. A simplificação do design dos kits de autoteste é uma recomendação frequente na literatura para melhorar a experiência do usuário e reduzir erros^(33)^.

Outro ponto importante é a dificuldade para interpretar os resultados (30%), um aspecto crítico para que o autoteste cumpra seu papel de informar e direcionar a conduta do usuário. A leitura de linhas visíveis ou fracas nos dispositivos pode gerar dúvidas, especialmente na ausência de um profissional de saúde. Estudos sugerem que a incerteza na leitura do resultado pode levar tanto ao falso sentimento de segurança quanto à ansiedade desnecessária^(34)^, tornando a experiência do autoteste desagradável para o usuário.

Por fim, os resultados deste estudo apontam para a necessidade urgente de incorporar a lente da equidade na formulação e implementação de tecnologias em saúde, especialmente aquelas voltadas ao diagnóstico autoadministrado. A baixa percepção de usabilidade identificada entre os participantes compromete o potencial dos autotestes como estratégia efetiva de saúde pública, particularmente em contextos de maior vulnerabilidade social e menor acesso a serviços formais de diagnóstico.

Embora os autotestes representem um avanço no enfrentamento de infecções como a COVID-19, sua efetividade está diretamente condicionada à capacidade dos usuários de compreender, executar e interpretar corretamente o procedimento. Isso demanda mais do que qualidade técnica no desenvolvimento dos produtos, exige um compromisso institucional com ações educativas, materiais instrucionais socioculturais adequados e suporte comunitário contínuo.

Nesse sentido, é imprescindível que políticas públicas voltadas à ampliação da testagem rápida não se restrinjam à distribuição dos autotestes, mas estejam acompanhadas de estratégias de suporte que promovam acessibilidade real, confiança no uso e autonomia dos usuários. No contexto do Sistema Único de Saúde (SUS), sustentado pelos princípios da universalidade, integralidade e equidade, torna-se essencial evitar a polarização tecnológica, que tende a beneficiar apenas grupos mais escolarizados ou com maior capital informacional e digital.

Popularizar tecnologias diagnósticas significa aproximá-las da realidade concreta da população brasileira, considerando níveis variados de letramento, escolaridade, idioma, cultura, condições de moradia e acesso à informação. Isso envolve investir em materiais visuais de fácil compreensão, dispositivos com design centrado no usuário e iniciativas locais que envolvam agentes comunitários de saúde, fortalecendo a ponte entre inovação e justiça social.

Dessa forma, os autotestes poderão cumprir, de fato, seu papel como instrumentos de cuidado, contribuindo para a vigilância epidemiológica, o autocuidado e o acesso equitativo ao diagnóstico em saúde.

### Limitações do estudo

O estudo apresenta algumas limitações que devem ser consideradas ao interpretar os resultados. Primeiramente, a amostra pequena pode limitar a generalização dos achados para outras populações. No entanto, a amostra permite uma análise exploratória robusta sobre a percepção de usabilidade do autoteste e suas possíveis associações com variáveis sociodemográficas, fornecendo subsídios relevantes para pesquisas futuras e para o aprimoramento de estratégias de testagem em populações vulnerabilizadas.

Além disso, o estudo foi realizado em um contexto restrito, o que pode não refletir as dificuldades e percepções de outros grupos populacionais em diferentes regiões ou com características socioeconômicas distintas. Por fim, não foi possível controlar variáveis como o nível de experiência prévia com autotestes ou com outros dispositivos de diagnóstico, o que pode ter impactado a facilidade de uso observada pelos participantes.

### Contribuições para a área

Nosso estudo evidencia a relevância de incorporar a perspectiva das iniquidades sociais na formulação e implementação de estratégias voltadas ao uso de autotestes diagnósticos, ao demonstrar que determinantes socioeconômicos, como a renda, influenciam a percepção de usabilidade. Ao identificar obstáculos relacionados à compreensão das instruções e ao manuseio dos insumos diagnósticos, a investigação fornece subsídios para o aprimoramento de materiais instrucionais e para a otimização do design voltado à usabilidade dos kits, com foco na acessibilidade e na segurança de populações em situação de vulnerabilidade social.

Adicionalmente, os achados apresentam aplicabilidade transdisciplinar, podendo ser estendidos ao desenvolvimento de orientações mais inclusivas para outras tecnologias em saúde utilizadas no contexto domiciliar, como esquemas terapêuticos autoadministrados, dispositivos de monitoramento remoto e estratégias de autocuidado. Dessa forma, o estudo contribui para o fortalecimento das práticas de cuidado centrado no usuário, para a qualificação do autocuidado supervisionado e para a promoção da equidade no acesso e na adesão a intervenções terapêuticas em ambientes extra-hospitalares.

## CONCLUSÕES

Apesar do potencial promissor dos autotestes de antígeno para COVID-19 como ferramenta de ampliação do diagnóstico em larga escala, os achados deste estudo revelam uma realidade um pouco mais complexa: a usabilidade percebida é, em geral, baixa. Apenas um terço dos participantes considerou o teste de fácil utilização, enquanto a maioria relatou dificuldades em diversas etapas do processo, da leitura das instruções à interpretação do resultado.

Além disso, entre os fatores investigados, apenas a renda elevada se mostrou significativamente associada à alta usabilidade. Essa associação indica que, mais do que uma questão técnica, a capacidade de utilizar um autoteste com autonomia pode refletir desigualdades estruturais.

Por fim, acreditamos que é urgente repensar o desenho e a implementação dos autotestes, não bastando apenas distribuí-los. É necessário garantir que as instruções sejam acessíveis em linguagem, formato e contexto; que haja apoio educativo nas comunidades; e que o desenvolvimento de tecnologias em saúde incorpore o princípio da equidade desde sua concepção.

## Data Availability

Os dados de pesquisa só estão disponíveis mediante solicitação.
